# Sustainable conversion of waste plastics to biofuel: Process insights and fuel characteristics

**DOI:** 10.1371/journal.pone.0354825

**Published:** 2026-07-31

**Authors:** Uchhwas Banik, Muhammad Nurul Huda, Mohammad Harun-Ur-Rashid, Razu Ahmmed, Md. Anowar Hosen, Mohammad Ismail

**Affiliations:** 1 Clean Energy & Carbon Capture Laboratory, Department of Applied Chemistry and Chemical Engineering, University of Dhaka, Dhaka, Bangladesh; 2 Air Quality and Environmental Pollution Research Laboratory, Centre for Advanced Research in Sciences (CARS), University of Dhaka, Dhaka, Bangladesh; 3 Department of Chemistry, International University of Business Agriculture and Technology (IUBAT), Uttara Model Town, Dhaka, Bangladesh; National Institute of Oceanography and Fisheries, EGYPT

## Abstract

Plastic consumption has become pervasive in modern society, with over 300 million metric tonnes produced annually worldwide, contributing significantly to municipal waste. In Bangladesh, where annual per capita plastic use has risen to 22 kg as of 2022, innovative solutions for managing plastic waste are urgently needed. This research introduces a novel approach to the pyrolysis of various plastics (PET, PVC, PP, HDPE) within a temperature range of 300 °C to 550 °C to produce pyrolytic bio-oil and biochar. We established optimal conditions for each plastic type—500 °C for PET, PVC, and HDPE, and 450 °C for PP—resulting in maximized yields of high-quality liquid oils (61.3% for PP and 47.23% for HDPE). Unique to this study, we innovatively adjust the pyrolysis process parameters to enhance the yield and quality of the derived bio-oils, tailored specifically to the types of plastics treated. The liquid products were characterized as predominantly consisting of C6–C16 hydrocarbons, aligning them closely with naphtha, gasoline, and diesel specifications, suitable for use as renewable fuels. Furthermore, our research applies FTIR and GC-MS analyses in a novel way to provide a detailed examination of these bio-oils, revealing significant quantities of paraffinic hydrocarbons in PP and olefins and naphthenes in HDPE, contributing to their potential fuel applications. The solid char byproducts were also comprehensively characterized using SEM and XRD, providing insights into their suitability for various industrial applications. This study not only demonstrates the potential of pyrolysis to transform waste plastics into valuable renewable energy resources but also advances the technological framework for sustainable waste management practices, marking a significant leap forward in the efficiency and application of plastic waste conversion technologies.

## 1. Introduction

Population growth, urbanization, environmental degradation, and increased energy use have depleted the sources of fossil fuels. Consequently, the pressing needs of global socioeconomic restructuring demand robust and enduring alternative energy sources [1]. Bangladesh is confronting several energy-related crises, such as depleting fossil fuel reserves, escalating power consumption, a rapidly growing population, and heightened industrial competition with developing countries, all of which exacerbate the degradation of air, soil, and water quality [2]. To address these challenges, Bangladesh, along with other nations, can leverage underutilized abundant natural resources such as biomass, solar, wind, hydro, tidal, and geothermal energy. The per capita energy consumption in Bangladesh is considerably lower than the global average, standing at 0.28 toe, which includes approximately 497 kWh of electricity. From 2010 to 2016, the annual energy use increased significantly by 5.5%, coinciding with rapid economic growth of about 6.3% during the same period. Subsequently, the annual increase moderated to 2.9% over the next five years [3]. Natural gas is the predominant energy source, accounting for 59%, while oil (18%), biomass (17%), and coal (4%) collectively contribute to over one-third of the total energy supply. In Bangladesh, more than 77% of the rural population relies on conventional biomass fuels for energy. Only 22% of rural residents have access to electricity, compared to about 32% nationally. Furthermore, only 3–4% of households are connected to natural gas for cooking. Over 90% of households depend on biomass for their energy needs, whereas only 2–3% use kerosene for the same purpose [4, 5].

Municipal solid wastes, including waste plastics, constitute a significant portion of fossil-based wastes that can be transformed into economically viable renewable fuels or chemicals with added value. Polyethylene (PE), polypropylene (PP), polyethylene terephthalate (PETE or PET), polystyrene (PS), nylons, and polyvinyl chloride (PVC) are the six primary types of plastics commonly utilized. These materials are contemporary and indispensable items in our daily household activities [6, 7]. They offer exceptional attributes, including reduced production costs, low weight, thermal stability, durability, and strength. As a result, they are used as alternatives to conventional materials such as wood, metals, grass, and ceramics [8]. Their slow disintegration rates, ranging from 100 to 1000 years, classify them as non-biodegradable and very harmful pollutants [9]. In 2013, the amount of waste plastics increased by 4% compared to 299 million tons in 2012, and it is expected to continue to rise [10]. According to the World Bank 2021 report, Bangladesh ranks as one of the leading countries in the world in terms of plastic pollution, primarily due to insufficient management of plastic waste. Annually, the rate of plastic consumption in Dhaka city is 22.25 kg per capita, which is three times higher than in other urban cities worldwide. Furthermore, the business consulting company LightCastle Partners reported that Bangladesh ranks 10th globally in the plastic industry. Every day in Dhaka, plastic waste has grown from 178 tonnes in 2005 to an astonishing 646 tonnes by 2020 [11].

Proper management and advanced technologies are crucial in mitigating issues related to municipal solid wastes, including waste plastics. Pyrolysis, a process involving the thermal decomposition of organic materials in the absence of oxygen, transforms used plastics into valuable products such as biochar, syngas, and bio-oil. This research innovatively employs a variety of reactor types, including fixed-bed, fluidized-bed, and spinning reactors, to optimize the pyrolysis process [12, 13]. Typically, the process operates at temperatures ranging from 300 to 800 °C, tailored to the specific material properties and desired product outcomes [14].The novelty of this study lies in its comprehensive approach to optimizing the pyrolysis conditions for various plastics like PET, PVC, PP, and HDPE, achieving maximum yields of high-quality liquid oils (e.g., 61.3% for PP at 450 °C). Through meticulous FTIR and GC-MS analyses, the research delves into the chemical properties of the bio-oils, confirming their suitability as renewable fuels due to their significant hydrocarbon content spanning the C6–C16 range. However, the broad compositional variability of plastic wastes presents challenges in standardizing the pyrolysis process, often leading to inconsistent product quality and yields. Impurities such as dirt, moisture, and non-plastic materials can adversely affect both the pyrolysis dynamics and the quality of the outputs, necessitating intricate and costly preprocessing steps [15]. Despite these challenges, the study advances the conversion technology by designing a continuous pyrolysis process that not only enhances the efficiency and quality of the outputs but also improves environmental performance. The primary objective of this research was to transform waste plastics into value-added biofuel, contributing to sustainable fuel security. By optimizing the plastic pyrolysis processes, the study not only addresses the immediate challenges of waste management but also enhances the economic and sustainability aspects of biofuel production from plastics. The implications of this work extend beyond academic and industrial realms, offering scalable and adaptable solutions for global waste management and energy production challenges.

## 2. Materials and methodology

### 2.1. Permits and field-site access

Waste plastic samples used in this study were collected from publicly accessible municipal solid waste collection points in Dhaka, Bangladesh. The samples consisted only of discarded post-consumer plastic materials, including PET, PVC, PP, and HDPE, and did not involve any protected areas, privately restricted sites, human participants, animals, endangered species, or biological specimens. Therefore, no formal environmental, ethical, wildlife, or field-collection permit was required for this work. Access to the waste collection sites was obtained with verbal permission from the relevant local waste-management personnel/site supervisors responsible for the municipal waste collection points. No personally identifiable information or sensitive data were collected during sample acquisition. The collected plastic wastes were manually sorted, washed, dried, and processed solely for laboratory-scale pyrolysis experiments.

### 2.2. Sample Collection and Feed Preparation

**Fig 1:**
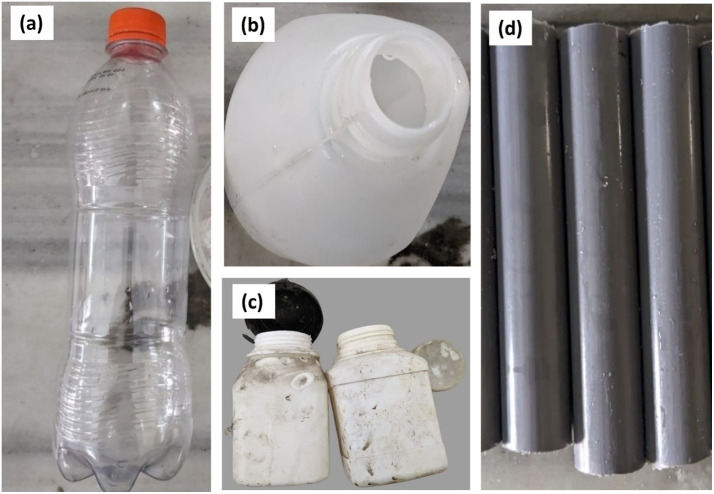
Collected waste plastics (a) PET, (b) PP, (c) HDPE, and (d) PVC.

The plastics were collected from the campus of Dhaka University and Old Dhaka (illustrated in Fig 1). The collected plastic waste was sorted into different categories such as PP, HDPE, PET and PVC before further processing. Mixed plastic waste was first manually sorted according to resin identification codes, product labels, physical characteristics, and visual appearance. To improve separation accuracy, density-based flotation techniques were applied where appropriate. Polyolefin plastics such as PP and HDPE, having densities lower than water (<1.0 g cm ^−^ ³), floated during water separation, whereas PET and PVC, possessing densities greater than water (>1.3 g cm ^−^ ³), sank. This density-based approach is widely used for preliminary segregation of mixed plastic waste and improves feedstock homogeneity prior to pyrolysis.

### 2.3. Experimental setup

#### 2.3.1. Pyrolysis plant.

A stainless-steel batch reactor, measuring 30 cm in both diameter and length, was employed for the pyrolysis process. This reactor was externally heated using an electrical system, equipped with an automatic temperature controller to ensure precise control of the heating elements. The reactor’s temperature was constantly monitored by a digital temperature controller. Integral components of the fixed-bed reactor include electrical heating elements to maintain the desired internal temperature, a water-cooled condensing coil that converts condensable vapor into pyrolytic liquid, and various other elements such as a temperature sensor, a heating coil, thermal insulation, a storage tank, a valve, and a gas exit line. Notably, the temperature sensor was strategically placed to gauge the temperature through the walls of the stainless-steel pyrolysis chamber.

The pyrolyzer is designed to operate within a temperature range of 400–600 °C, adjustable in 50 °C intervals. Thermocouple sensors, protruding inside the reactor chamber, facilitated continuous temperature monitoring. Each experimental run began by manually loading 100 grams of the sample into the reactor. Prior to initiating the experiment, nitrogen gas (N_2_) was flowed through the reactor for two minutes to purge any residual air, ensuring a controlled and oxygen-free environment. The preheater was also initially filled with N_2_ gas. Subsequently, the reactor heater was activated, gradually raising the temperature to the target of 400 °C, as indicated by the temperature display.

During the experiments, temperatures were meticulously recorded using the digital display. A steady supply of nitrogen gas was maintained to preserve the inert atmosphere within the reactor and to efficiently sweep the pyrolyzed vapor towards the condensers. This vapor was then condensed into pyrolytic liquid, collected in bottles positioned beneath the condensers. Any gases that did not condense were safely flared into the atmosphere. The collection bottles were removed once they were filled with liquid, ensuring they were free from any air bubbles. Upon completion of the decomposition process, a colorless gas was emitted, signaling that the thermal decomposition of the plastic sample was complete. The absence of color in the flare indicated thorough pyrolysis.

Once the pyrolysis was completed, the reactor heater was switched off, the vapor exit was sealed, and the nitrogen supply was discontinued. The char byproduct was then removed from the reactor chamber after it had cooled down and was subsequently weighed. The weight of uncondensed gases was calculated by subtracting the total weight of the feedstock from the combined weight of the liquid and char. Finally, the system was reset and prepared for the next run, ensuring a seamless continuation of the pyrolysis process.This meticulous setup and process underline the controlled environment necessary for efficient pyrolysis, aiming to maximize the yield and quality of the bio-oil and char, thereby contributing effectively to waste management and resource recovery.

**Fig 2:**
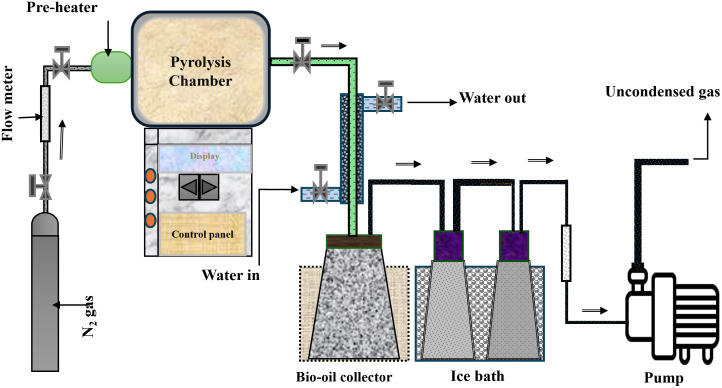
Experimental setup of the pyrolysis plant, showing reactor, temperature control, nitrogen purging, and bio-oil condensation.

Fig 2 illustrates the experimental pyrolysis setup used to convert waste plastics into bio-oil, biochar, and gaseous products. The setup features a stainless-steel batch reactor with a 30 cm diameter and length, equipped with electrical heating elements, a digital temperature controller, and a nitrogen gas purging system to maintain an oxygen-free environment. The pyrolysis vapors generated within the reactor are directed to a water-cooled condenser, where they are converted into liquid bio-oil. Non-condensable gases are safely flared, and the solid char is collected from the reactor after cooling. This setup ensures precise temperature regulation and efficient collection of pyrolysis products.

#### 2.3.2. Distillation unit.

Fractional distillation, also known as fractionation, represents a specialized distillation technique that significantly surpasses traditional distillation in terms of efficiency and effectiveness. This process relies on multiple distillation cycles or repeated vaporization-condensation cycles, making it akin to conducting a series of distillations consecutively. Each cycle achieves a hypothesized equilibrium stage, and for effective separation of the vapor or liquid mixture, it might be necessary to execute several such theoretical steps. In this intricate setup, the burner used for the distillation is fueled by methane gas, providing a consistent and controlled source of heat. The distillation itself is conducted using a 500 ml round bottom flask, which is part of a distillation column that stands 40 cm tall. This setup is designed according to specific rules of thumb that provide general guidance, although these guidelines often have exceptions due to the varying conditions under which distillation might occur. These guidelines include crucial parameters for the design of the distillation column. The length-to-diameter ratio of the column is advised to be less than 30, ideally below 20, to ensure structural integrity and operational efficiency, particularly because exceeding a height of 60 meters can lead to issues related to wind load and foundational stability. If the tower height exceeds 60 meters, it is recommended to consider a design with smaller tray spacing to accommodate for the increased structural demands [16]. Furthermore, the ratio of the tower’s diameter to the random packing size should be greater than 10. For optimal vapor disengagement, the diameter at the top of the tower is maintained at 1.2 meters, ensuring a smooth and efficient separation process. These methodical approaches in the design and operation of fractional distillation systems underscore their critical role in achieving precise separation and purification outcomes in chemical processing and experimental setups. This rigorous approach to distillation design and operation, guided by foundational principles and specific industry guidelines, exemplifies the advanced techniques used in modern chemical engineering [17].

In this experimental setup, a small-scale thermometer calibrated in degrees Celsius was securely mounted in the distillation column using a thermometer holder. This arrangement ensured accurate temperature readings necessary for precise control of the process. The thermometer was held firmly in place with M-seal gum, which provided a stable and appropriate positioning to yield reliable results. For the condensation process, a Liebig condenser was utilized, through which tap water was circulated at a moderate flow rate in a counter-current manner. This setup effectively condensed the vapor within the system. The condenser, measuring approximately 70 cm in length, played a critical role in the efficient cooling and condensation of vapors to liquid form. A specialized oil collector was employed to separate and collect different oil fractions. This collector was designed to allow the escape of non-condensable gases, ensuring that only the desired liquid fractions were retained. To enhance thermal efficiency, glass fibers were processed into a wool-like structure using a binder, forming glass wool—an insulating material renowned for its thermal properties. The creation of glass wool involves a technique that generates numerous tiny air pockets between the glass fibers, significantly enhancing its thermal insulation capabilities. Available in slabs or rolls, glass wool can be adapted for various applications. It is often either sprayed on or applied directly to surfaces requiring insulation, providing both mechanical strength and thermal resistance [18].

### 2.4. Pyrolysis Process

**Fig 3:**
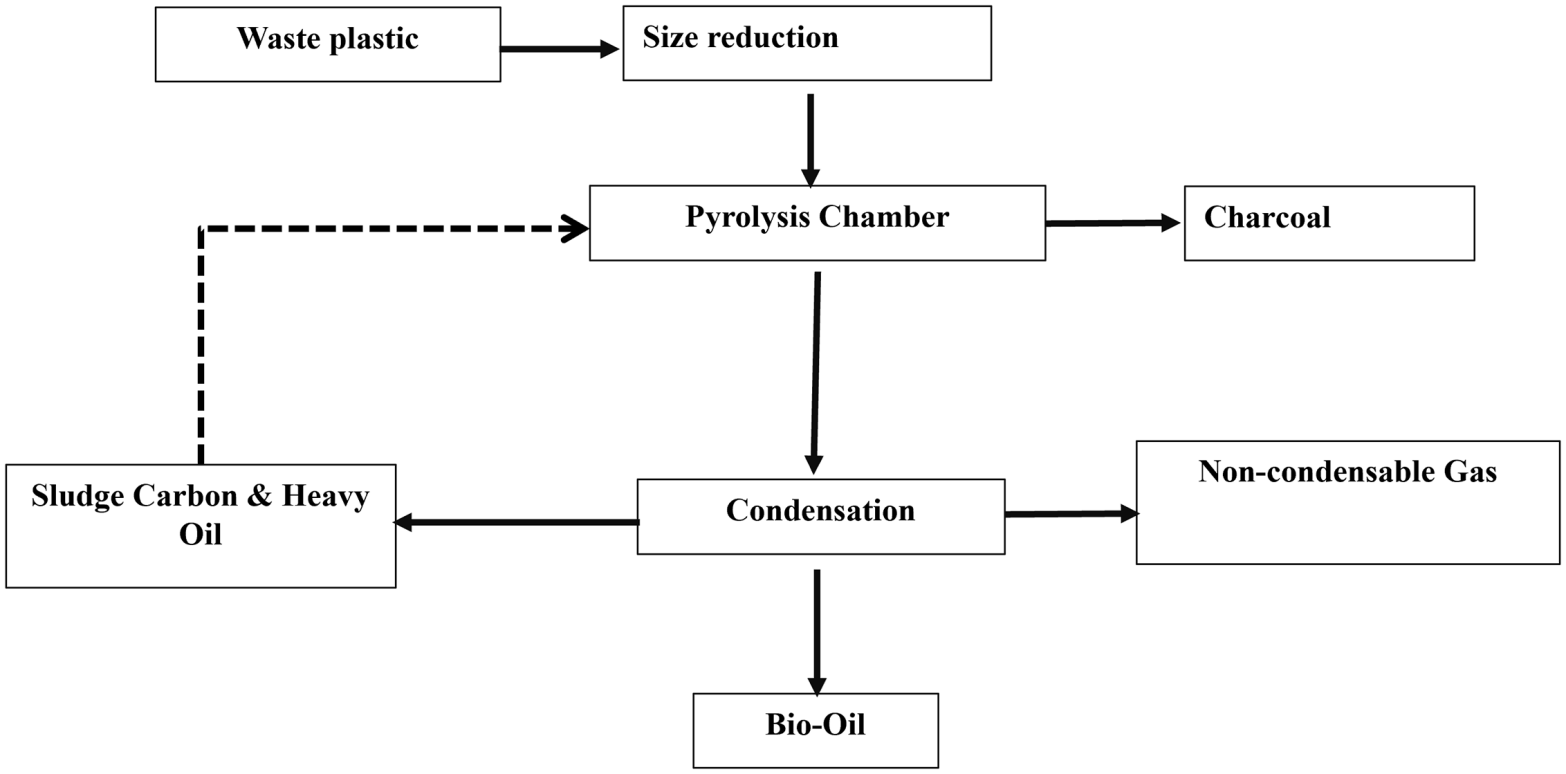
Schematic of the proposed process flow diagram.

Before the pyrolysis process commenced, the plastic chips were meticulously cleaned and dried. These chips were then heated outside the reactor in an oxygen-free environment to prevent combustion. During pyrolysis, the large molecules of the liquefied polymers were transformed into steam, rising out of the reactor at high temperatures. This vapor was then directed to a reactor condenser, specifically designed to cool and condense the vapor back into a liquid form. The process was maintained for 60 minutes within a temperature range of 400–550 °C, ensuring optimal conversion of the polymers. As the pyrolysis byproducts emerged as vapor, they were channeled into a water-cooled condenser where they condensed into a liquid. This liquid, still unrefined, was collected and could potentially be used as fuel. The system was designed to release any gases that did not condense into the atmosphere, minimizing pressure build-up within the apparatus. The operation of this process is illustrated in Fig 3, which provides a schematic overview of the waste plastics pyrolysis setup. The oil collected from this process was derived from the described pyrolysis plant, capturing the essence of transforming waste into valuable resources through controlled thermal degradation.

### 2.5. Physiochemical and elemental analysis

The physicochemical and elemental analyses of the pyrolytic oil derived from waste plastics were conducted to comprehensively characterize its properties and potential as a biofuel. These analyses involved the determination of key properties such as density, viscosity index, flash point, pour point, and ash content. The tests were carried out using a variety of specialized instruments while adhering strictly to the protocols outlined by the Institute of Petroleum (IP) and the American Society for Testing and Materials (ASTM) standards. These standards ensure that the results are reliable and comparable to conventional fuels.

Density and viscosity measurements are critical for understanding the flow characteristics and energy content of the pyrolytic oil, influencing its suitability for various combustion applications. The flash point and pour point assessments provide insights into the safety and usability of the oil under different temperature conditions, particularly in cold climates. The ash content analysis is vital for evaluating the cleanliness of the fuel during combustion, with lower ash content indicating fewer residues and emissions. These analyses collectively provide a detailed understanding of the pyrolytic oil’s properties, allowing for a comparison with conventional fossil fuels. The results from these evaluations support the feasibility of using pyrolytic oil as a renewable energy source, contributing to the broader goal of sustainable waste management and energy production.

### 2.6. Characterization techniques

The compositional analysis of the plastic pyrolytic oil was carried out in the form of functional group and the characteristics of the molecule were measured from its absorbance spectrum. The functional group compositions of the PPO (plastic pyrolysis oil) were analyzed by Fourier transform infra-red spectroscopy (FTIR) to identify the basic compositional groups. The IR-spectra of the plastic oil was produced by a standard FTIR instrument (model SHIMADZU FTIR 4500) and an online pen plotter. Information about the composition and type of organic compounds of bio-oil and its distillate product was obtained by Gas Chromatography/Mass-Spectroscopy (GC/MS) analysis by *GC-MS-QP 2010 Ultra* using a capillary column (30 × 0.25 mm × 0.25 μm). The XRD patterns of the materials were examined using an X-ray diffractometer (Ultima IV, Rigaku Corporation, Akishima, Japan) fitted with Cu K radiation (= 0.154 nm). Scanning electron micrographs were acquired from JEOL-JSM 5600 LV microscope with a 6587 EDS (energy dispersive X-ray spectrometry) detector.

## 3. Results and discussion

Pyrolysis produces three main byproducts, including pyrolytic oil, gas, and char. Among these, the most significant is the crude oil, which has been thoroughly analyzed in this study. The yield and quality of these products are influenced by factors such as temperature, time, pressure, and reactor design. Pyrolysis involves breaking down large hydrocarbon chains under specific temperatures, times, and pressures in the absence of oxygen, resulting in solid, liquid, and gaseous products. At 550 °C, the process primarily yields a liquid consisting of various hydrocarbons depending on the waste material’s initial composition. The optimal temperature for maximizing oil production is within the range of 400 °C to 550 °C; beyond 550 °C, gas formation dominates due to increased liquid cracking. Methane (CH_4_) is the main component of the gas, along with smaller amounts of C_2_H_6_, C_2_H_4_, C_2_H_2_, and other gaseous hydrocarbons. The reactor’s design and temperature play crucial roles in determining the quantity and quality of the products. In this study, vacuum pyrolysis was utilized to recover oil from waste plastics. Although the process also generates solid black carbon and pyrolysis gas, the primary focus is on the liquid output.

### 3.1. Product analysis

In the plastic pyrolysis system, three primary products are obtained: liquid pyrolytic oil, char, and non-condensable gases. Among these, pyrolytic oil is the main product of interest in the present study because of its potential application as a renewable fuel. Char and non-condensable gases may also be used as supplementary energy sources for process heating, thereby improving the overall energy efficiency of the pyrolysis system. Multiple experimental runs were conducted using prepared plastic waste samples under controlled pyrolysis conditions. The pyrolysis process involves the thermal decomposition of long-chain plastic polymers into smaller hydrocarbon compounds through complex cracking and free-radical reactions. The product distribution is strongly influenced by plastic type, temperature, reactor design, residence time, and heating conditions.

The overall decomposition pathway of plastic waste during pyrolysis can be represented as follows:

Decomposition of polymers:


$$\begin{array}{c} \text{Plastic polymer }\to \text{ Pyrolytic oil }+\text{ Non}-\text{condensable gas }+\text{ Char} \end{array}$$

Cracking of heavy hydrocarbons:


$$\begin{array}{c} \text{Heavy hydrocarbons }\to \text{ Light hydrocarbons }+\text{ Non}-\text{condensable gases} \end{array}$$

Expected gas-phase products:

$\text{Non}-\text{condensable gas }\to {\text{H}}_{\text{2}}+\text{ C}{\text{H}}_{\text{4}}+{\text{C}}_{\text{2}}{\text{H}}_{\text{6}}+{\text{C}}_{\text{2}}{\text{H}}_{\text{4}}+{\text{C}}_{\text{2}}{\text{H}}_{\text{2}}+\text{ CO }+\text{ C}{\text{O}}_{\text{2}}$

These equations represent simplified pyrolysis pathways based on typical thermal cracking behavior of plastic polymers. In the present study, the total gaseous product yield was estimated by mass balance; however, individual gaseous species were not quantified experimentally.

The gas phase generated during plastic pyrolysis is expected to contain light gases such as H_2_, CH_4_, C_2_H_6_, C_2_H_4_, C_2_H_2_, CO, and CO_2_, depending on the feedstock type and operating conditions. The pyrolytic oil contains a mixture of light to heavy hydrocarbons, and its composition varies according to the plastic material processed. Notably, HDPE and PP produced a substantial amount of liquid hydrocarbons within the C₆–C₁₆ range, making these oils potentially suitable for refining into fuel-range products. The char produced during pyrolysis is a carbon-rich solid residue and may have potential applications as a solid fuel or as a precursor for activated carbon after suitable post-treatment. Table 1 highlights the distinct product yields and characteristics obtained from the pyrolysis of different plastic materials. The optimized conditions show that PP and HDPE provide the highest yields of pyrolytic oil, supporting their potential use in renewable fuel production.

**Table 1:** Pyrolysis product mass balance and characteristics of different plastic materials.

Materials	Pyrolysis Gas (%)	Pyrolysis Oil (%)	Char (%)	Key Characteristics	Optimal Temperature Range (°C)
**PET**	69.94	16.21	18.85	High gas yield, low oil yield due to aromatic structure	500–550
**PVC**	56.64	9.00	34.36	High char content due to chlorine, challenging to process	450–500
**HDPE**	46.05	47.23	6.72	High oil yield, naphtha and diesel range hydrocarbons	450–500
**PP**	33.49	61.30	5.21	Highest oil yield, rich in paraffinic hydrocarbons	400–450

The char yields from PP and HDPE were 5.21% and 6.72%, respectively, indicating that both polyolefin plastics generated relatively low solid residues compared with PET and PVC. However, the XRD patterns suggest that the structural and inorganic residue characteristics of these chars were not identical.

Hydrogen formation in the non-condensable gas fraction:

Hydrogen may be produced during plastic pyrolysis through secondary cracking, dehydrogenation, and free-radical reactions of hydrocarbon fragments at elevated temperatures. In the present study, H_2_ is considered a possible component of the non-condensable gas fraction together with CH_4_, C_2_H_6_, C_2_H_4_, C_2_H_2_, CO, and CO_2_. However, the gas fraction was not collected for detailed compositional analysis using GC-TCD, GC-FID, or other gas analyzers. Therefore, the exact concentration or yield of H_2_ could not be quantified in this work. The total gaseous product yield was estimated by mass balance, but individual gas components were not measured separately. Accordingly, H_2_ production is discussed qualitatively rather than quantitatively. Future studies should include gas-phase analysis to determine the precise H_2_ content and evaluate the possible use of pyrolysis gas as a hydrogen-containing energy stream.

### 3.2. Thermal cracking of PP and HDPE

In the thermal cracking of PP and HDPE, these polyolefin polymers serve as representative models in pyrolysis research to explore the impact of temperature on the development of various cracking products. The pyrolysis process was investigated in a nitrogen environment across a range of temperatures, including 250 °C, 300 °C, 350 °C, and 400 °C. The pyrolysis reaction occurs through three successive phases: initiation, propagation, and termination. During the initiation phase, large polymer molecules undergo thermal cleavage, resulting in the formation of free radicals. In the propagation phase, these free radicals and molecular species continue to break down into smaller radicals and molecules. Finally, in the termination phase, the unstable free radicals are converted into stable compounds.

The mechanism of the free radical route and random chain scission of thermal cracking involves several key steps. During the initiation phase, large polymer molecules undergo thermal cleavage, leading to the formation of free radicals. In the propagation phase, these free radicals react with the polymer chains, causing further breakdown into smaller molecules and additional free radicals. The termination phase involves the stabilization of these radicals into stable end products, which can include aliphatic hydrocarbons, olefins, and gases like hydrogen. The process can be described in detail as follows:

Initiation (Thermolysis):

Large polymer chains (e.g., PP, HDPE) are subjected to thermal energy, leading to the homolytic cleavage of carbon-carbon bonds, producing primary free radicals.


$$\begin{array}{c} \text{R}-\text{R }\to \text{ 2R}\cdot \end{array}$$

Propagation:

The primary free radicals react with the polymer chains, causing further breakdown. This reaction can proceed through hydrogen abstraction, where a radical abstracts a hydrogen atom from a neighboring polymer, generating a new radical and a stable molecule.


$$\begin{array}{c} \text{R}\cdot +\text{ R}-\text{H }\to \text{ R}-\text{H }+\text{ R}\cdot \end{array}$$

Alternatively, a radical may undergo β-scission, where it fragments to form a smaller radical and a stable molecule.


$$\begin{array}{c} \text{R}\cdot \to {\text{R}}^{\prime }+\text{ R}\cdot \end{array}$$

Termination:

The free radicals combine to form stable molecules. This can occur through several pathways, such as recombination or disproportionation, where two radicals combine to form a stable product.


$$\begin{array}{c} \text{2R}\cdot \to \text{ R}-\text{R} \end{array}$$

This study reveals that the cracking of polypropylene (PP) and high-density polyethylene (HDPE) is strongly temperature-dependent, with distinct product distributions emerging at varying temperatures. As temperatures rise, there is a notable shift towards the production of lighter hydrocarbons and gaseous products, underscoring the critical role of temperature control in optimizing the yield of targeted products. By delving into free radical mechanisms and random chain scission pathways, the research provides valuable insights that enhance the efficiency and yield of the pyrolysis process, contributing significantly to plastic waste management and renewable energy generation. Building on existing research, this investigation introduces novel approaches to refining pyrolysis conditions specifically for polyolefin polymers. The study’s rigorous analysis and systematic methodology make its findings both authoritative and transformative, offering groundbreaking insights that are likely to drive future advancements in the field.

### 3.3. Effect of temperature on product yield

The study initially focused on temperatures of 400 °C and 550 °C, as these were identified as crucial for significant thermal degradation based on TG and DTG data. A response time of 60 minutes was selected to ensure complete thermal degradation at these temperatures. To optimize the liquid yield from the raw material conversion, subsequent tests varied the reaction time between 30 and 90 minutes. The research examined the influence of reactor temperature on pyrolysis production across a range of 400 °C to 550 °C. The choice of 400 °C as the starting temperature was driven by the observed degradation rates. However, at 400 °C for 60 minutes, raw material conversion was minimal, resulting in a solid residue of nearly 40% (39.86%). Since complete thermal degradation of all plastic materials occurred below 550 °C, this temperature was chosen as the final process temperature. Multiple TG and DTG analyses corroborate this temperature range by highlighting the peak thermal degradation rates of the polymers within this interval [19, 20].

**Fig 4:**
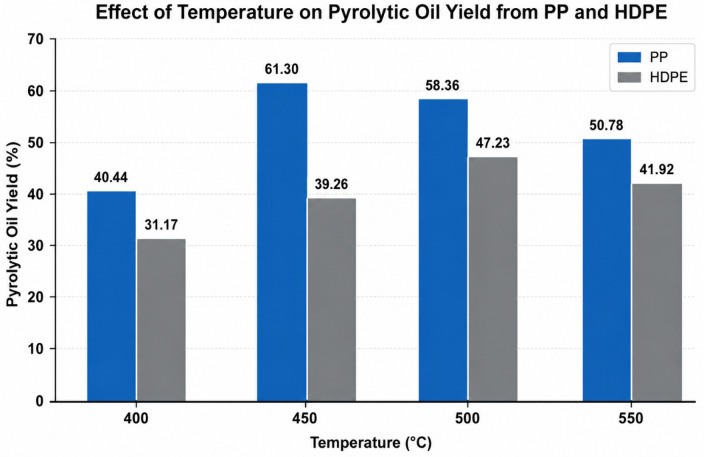
Pyrolysis of PP and HDPE at different temperature.

Initial experiments were conducted at 400 °C and 550 °C due to the significant thermal deterioration velocities observed at these temperatures in TG and DTG data. A 60-minute response time was employed to achieve total thermal degradation at the indicated temperature. Further tests adjusted the reaction time between 30 and 90 minutes to maximize the liquid yield from the raw material conversion. The effects of reactor temperature on pyrolysis production were studied across temperature intervals of 400 °C to 550 °C. The increased deterioration rates observed at 400 °C justified its selection as the starting temperature. However, at 400 °C for 60 minutes, raw material conversion remained minimal, with a solid residue of 40% (39.86%). Complete thermal degradation of all plastic materials was achieved below 550 °C, validating this as the final process temperature. TG and DTG analyses of the polymers’ peak thermal breakdown rates further support this temperature interval [19, 20]. Fig 4 illustrates how reactor temperature affects the pyrolysis production of the samples studied, showing that even at 400°C, a significant amount of raw material remains unreacted, leading to the decision to not conduct studies at lower temperatures.

The rate of raw material conversion increased with temperature, with HDPE and PP producing the highest liquid phase (pyrolysis oil) yields at 500 °C and 450 °C, respectively. As the temperature increased, gaseous products increased while pyrolysis oils decreased due to higher temperatures favoring secondary reactions that cause chain-cracking and the formation of shorter molecules or non-condensable gases. This behavior is consistent with observations from other researchers. Ayhan Demirbas reported that the gaseous product yield of a plastic mixture (PP, PE, and PS) increased from 12.3% to 42.4% as the temperature rose from 550 to 900 K. The liquid product output increased up to 750 K (477°C) before declining significantly up to 900 K [21]. According to Achilias et al. [22], the pyrolysis of PP, LDPE, and HDPE in a fixed bed reactor at 450 °C for 17 minutes resulted in low conversion rates, even with a small sample size of 0.7 g, leaving substantial solid residues for LDPE (76.4%), HDPE (76.7%), and PP (46.6%) [22]. However, a combination analysis of PP and HDPE at the same temperature with a different reaction time of 45 minutes showed less residue (Fig 4), suggesting that reaction time significantly influences conversion. Saad & Williams [23] found that 80% of the products were condensable when synthetic and waste PP were pyrolyzed in a fixed bed reactor at 500 °C using a 20 g sample [23]. On the other hand, Adrados et al. [24] and López et al. [25] observed complete conversion at 460°C from a mixture of pure synthetic polymers (PP 40%, PE 35%, PS 18%, PVC 3%, and PET 4%) in a pyrolysis reactor [24, 25]. For superior quality products, a temperature of 500°C was recommended, yielding 65.2% liquid product. The optimal reactor temperatures for maximal liquid product yield in the tested laboratory pyrolysis setup with a fixed bed and typical heating of the plastic material combination were 500°C for HDPE and 450 °C for PP within 45 minutes.

### 3.4. Temperature distribution of plastic pyrolysisplant

**Fig 5:**
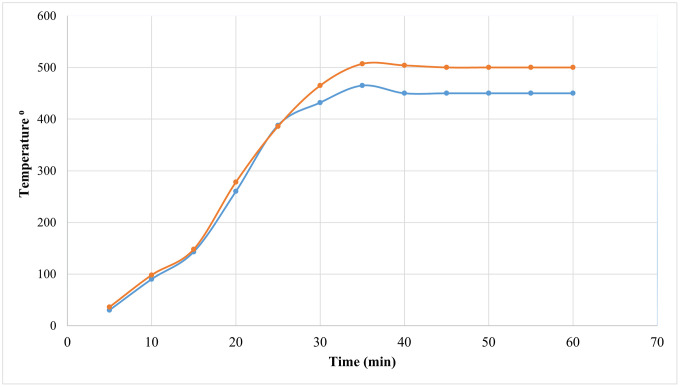
Temperature distribution in reactor with time.

The temperature distribution within a pyrolysis plant plays a crucial role in determining the efficiency and yield of the process. Initially, the reactor temperature is maintained between 20–25°C, which is the ambient temperature. As the reactor is heated, temperature readings are recorded at five-minute intervals. The heating process is gradual, with the reactor temperature steadily increasing during the first 30–40 minutes of operation. Following this, the reactor is maintained at a constant temperature of approximately 450 °C (±10 °C) and 500 °C (±10 °C) for the next 30–40 minutes. This consistent temperature distribution is critical to ensuring uniform pyrolysis and optimal product yield. The recorded temperature data, as depicted in Fig 5, shows that the reactor temperature stabilizes at around 450 °C and 500 °C, which are the key operational temperatures for the pyrolysis process. These temperature ranges are selected based on their effectiveness in breaking down the plastic feedstock into pyrolytic oil, gas, and char, thus maximizing the production of valuable byproducts.

### 3.5. Effect of time on product yield

**Fig 6:**
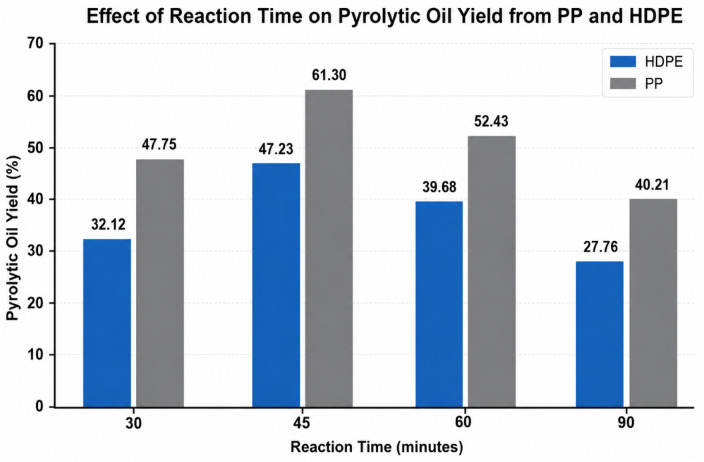
Pyrolysis of PP and HDPE at different time.

The study focused on evaluating the impact of reaction time on product yield during the plastic pyrolysis process, with a fixed temperature of 500 °C. This temperature was selected based on recommendations from previous studies by Adrados et al. and López et al., which emphasized the significance of 500 °C for investigating the influence of reaction time on plastic pyrolysis [24, 25]. The experiments varied the reaction time from 30 to 90 minutes, utilizing a nitrogen flow rate of 50 ml/min and a heating rate of 20 °C/min. The results, illustrated in Fig 6, demonstrate that a reaction time of 45 minutes achieved the maximum conversion of plastic into gaseous and liquid-condensable compounds. During the initial 45 minutes, the production of pyrolytic oil increased significantly, reaching a peak yield. However, extending the reaction time to 60 and 90 minutes resulted in a rapid decline in oil production. The highest yields of condensable products, 47.23% from HDPE and 61.30% from PP, were obtained at 45 minutes at 500 °C. Consequently, a reaction time of 45 minutes at this temperature is deemed sufficient for optimal conversion of raw materials in this particular reactor setup.

PP and HDPE were employed as representative polyolefin polymers in the pyrolysis study to explore the effects of temperature on the development of various cracking products. The pyrolysis process was investigated at temperatures of 250 °C, 300 °C, 350 °C, and 400 °C in a non-sweeping nitrogen atmosphere. The pyrolysis reaction occurs through three distinct phases: initiation, propagation, and termination. During the initiation phase, large polymer molecules undergo thermal cleavage, resulting in the formation of free radicals. In the propagation phase, these free radicals, along with molecular species, continue to break down into smaller radicals and molecules. Due to their instability, the free radicals undergo termination reactions, leading to their incorporation into stable molecules. The mechanism of the free radical pathway and random chain scission involved in thermal cracking is illustrated earlier in the manuscript.

Previous studies have cited a reaction time of 30 minutes for the pyrolysis of a mixture containing PP, PE, PS, PVC, and PET in a 3.5 L fixed bed reactor, with typical plastic mixture heating through the reactor wall [25]. In other laboratory reactors, such as the Parr Mini Bench Top Reactor (Type 4561 m), an autoclave with a volume of 300 ml and a mixer operating under 19.5 MPa pressure was used to process a waste plastic mixture (HDPE, PP, PS, PET, and PVC) at 500 °C for 60 minutes, although the effect of reaction time was not specifically examined [26]. The study highlights the differences between laboratory micro-reactors and pilot reactors, particularly regarding heat transfer mechanisms. Pilot reactors, while capable of processing small samples, have more restricted heat transfer mechanisms compared to laboratory micro-reactors. This limitation affects the efficiency of raw material conversion. Further studies are necessary, especially in pilot reactors, to better understand these heat transfer dynamics and optimize the pyrolysis process at a larger scale.

### 3.6. Fractional distillation of crude pyrolysis oil

**Fig 7:**
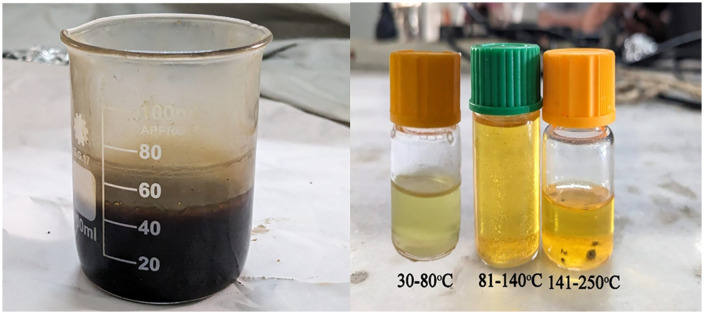
Crude Pyrolysis oil and fractionated liquid products from pyrolysis crude oil.

The fractional distillation of crude pyrolysis oil was carried out using a distillation column, effectively separating the pyrolytic liquid into distinct components based on their boiling points. The pyrolytic liquid was fractionated into five main categories: 30–80 °C, 81–140 °C, 141–180 °C, 181–250 °C, and a non-fractionated residual oil. Multiple successful runs were conducted using raw pyrolysis liquid samples to ensure an adequate yield of each fractionated oil component. The product distributions obtained through this method are depicted in Fig 7, demonstrating the efficiency of the fractional distillation process in refining the crude pyrolysis oil. This separation technique is crucial for enhancing the quality and usability of the derived products, aligning them more closely with conventional fuels. This approach not only refines the crude oil but also enables the isolation of valuable fractions that can be further processed or used directly, thus contributing to the overall efficiency and sustainability of the pyrolysis process.

### 3.7. Fuel Properties of plastic derived pyrolysis

The fuel properties of plastic-derived pyrolysis oil can vary widely due to the complex composition of the oil, which includes a mixture of different hydrocarbons. Key parameters such as density, viscosity, calorific value, flash point, pour point, and ash content are critical in determining the suitability of pyrolysis oil for various applications. While minor variations in these properties may have little impact on some uses, they can pose significant challenges when using pyrolysis oil in devices designed for conventional hydrocarbon fuels. These challenges may make the operation of such devices difficult or, in some cases, entirely unfeasible. As a result, it is essential to thoroughly characterize any fuel oil intended for use in engines and compare its properties with those of conventional diesel and other standard oils. Table 2 presents a comparative study of the properties of pyrolysis oil alongside those of conventional oils, highlighting the differences and similarities that influence its potential applications.

**Table 2:** Comparative study of pyrolysis oil obtained from PP and HDPE with other conventional oil.

	PP	HDPE	Gasoline	Diesel
Density g/cm^3^	0.81	0.84	0.78	0.80
Viscosity (mm^2^/s)	4.23	5.48	1.17	1.9-4.1
Flash Point (°C)	32	50	42	52
Pour Point (°C)	−10	−4	0	6
Calorific Value (MJ/kg)	25	26.5	44-46	42-46
Ash Content (%wt)	0	0	0	0.01

The densities of the liquid oils derived from PP and HDPE were measured at 0.81 g/cm^3^ and 0.84 g/cm^3^, respectively, aligning closely with the density range of diesel hydrocarbons (C12-C16). The viscosity of a fluid, whether Newtonian or not, reflects its resistance to shearing forces during flow and its ability to flow under gravity, which are crucial for the functionality of fuel injection systems. This characteristic also affects the fuel’s combustibility and atomization quality. The kinematic viscosity of the oil derived from PP was 4.23 mm^2^/s, while the HDPE liquid exhibited a viscosity of 5.48 mm^2^/s, both within the range required for high-quality gasoline and diesel fuels [27]. The flash point, an important safety parameter that defines the lowest temperature at which the vapors above a liquid ignite when exposed to an external flame, was recorded at 32 °C for the PP-derived liquid and 50 °C for the HDPE-derived liquid. These values are comparable to those of light petroleum distillate fuels. The pour point, which represents the lowest temperature at which a liquid ceases to flow and thereby indicates the fuel’s fluidity at low temperatures, was found to be –10 °C for the PP-derived liquid and –4°C for the HDPE-derived liquid. Although pour points for light distillate petroleum fractions vary, the pour point of naphtha-range fractions generally occurs around –6°C. The calorific values of the pyrolysis oils from PP and HDPE were determined to be 25 MJ/kg and 26.5 MJ/kg, respectively. While these values are slightly lower than those of conventional diesel fuel, they highlight the potential for using plastic-derived oils as a viable fuel source. Lastly, the ash content of each pyrolysate was negligible for both PP and HDPE-derived liquids, indicating the absence of metal contamination and high molecular weight soot, which further underscores the purity and quality of the liquid fractions.

### 3.8. Compositional Analysis

#### 3.8.1. FTIR.

**Fig 8:**
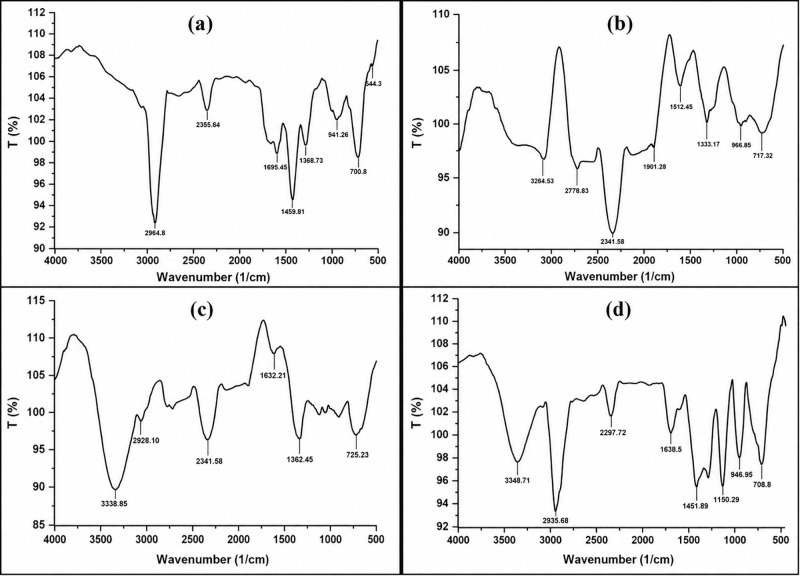
FT-IR spectra of, (a) raw PP, (b) PP pyrolysis oil, (c) Raw HDPE and (d) HDPE pyrolysis oil.

The FTIR spectra of raw plastic materials including PP, pyrolysis oil from PP, and HDPE, as well as pyrolysis oil from PP and HDPE, are presented in Fig 8. The functional group and composition analysis of the pyrolytic oil at different wave numbers per cm are detailed in S1–S4 Table (supporting information). The FTIR analysis revealed that the pyrolysis oil contains a variety of functional groups, predominantly alkanes, aromatics, alkenes, ketones, alcohols, and aldehydes. Specific absorbance peaks were observed: C-H stretching between 3000–2850 cm ^−^ ¹ and C-H bending between 1000–650 cm ^−^ ¹, indicating the presence of alkanes; C = C stretching between 1035–845 cm ^−^ ¹ and CH_2_ bending at 725.23 cm ^−^ ¹, further confirming the presence of alkanes. Additionally, alcohol groups were identified through O-H stretching between 3200–3400 cm ^−^ ¹ and C-O stretches between 1250–1300 cm ^−^ ¹. Aldehyde groups were also detected, with an absorbance peak for C = O stretching between 1850–1650 cm ^−^ ¹. The functional groups identified in the pyrolytic oil are quite similar to those found in diesel oil, as reported in other studies. The presence of hydrocarbon groups such as C-H, C = C, and alcohols suggests that the pyrolysis liquid has significant potential for use as a fuel.

#### 3.8.2. GC-MS.

GC-MS analysis was employed to determine the distribution of hydrocarbons with varying carbon chain lengths in the liquid products derived from PP and HDPE. The hydrocarbon distribution in the PP-derived liquid fraction, as shown in Table 3, was found to be 14.66%, 34.92%, 12.15%, 25.39%, and 7.60% across different carbon chain ranges. The significant proportions of hydrocarbons in the C13 to C16 (34.92%) and C17 to C20 (25.39%) ranges suggest that the liquid product is diesel-enriched. Nearly 50% of the product comprised light naphtha hydrocarbons (C6-C16).

**Table 3:** Products distribution in liquid fractions derived from PP and HDPE.

Distribution of hydrocarbon range products (% age)					
Feed Materials	C6-C12	C13-C16	C17-C20	C20 to C30	>C30
PP	14.66	34.92	12.15	25.39	7.60
HDPE	33.45	31.28	15.64	11.93	11.24

**Fig 9:**
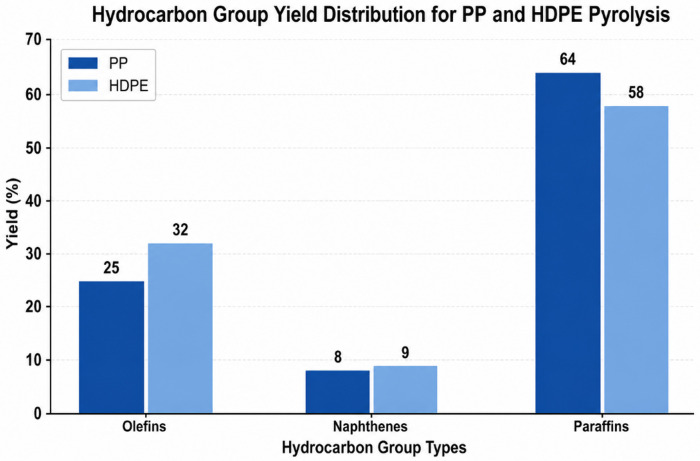
Hydrocarbon group type distribution in liquid fractions derived from PP and HDPE.

For the HDPE-derived liquid fraction, the hydrocarbon distribution was 33.45%, 31.28%, 15.64%, 11.93%, and 11.24% across the C6-C12, C13-C16, C17-C20, C20-C30, and >C30 ranges, respectively. This distribution indicates that the fraction is enriched with low boiling naphtha hydrocarbons. Diesel and gasoline hydrocarbons accounted for 63% of the total product, while C17 to C30 hydrocarbons constituted 34% of the liquid component. **S5****–****S6** Tables (supporting information), and Fig 9 illustrate the relative amounts of olefins, cycloalkanes, aliphatic, and aromatic components in the liquid products derived from PP and HDPE. The PP-derived liquid products contained 25% olefins, 7% cycloalkanes (naphthenes), and 66% paraffins, whereas the HDPE-derived liquid products consisted of 31% olefins, 8% cycloalkanes, and 59% paraffins.

Notably, PP produced a higher proportion of paraffinic compounds compared to HDPE, which resulted in a greater yield of olefins and naphthenes. Since paraffinic hydrocarbons possess higher energy content than aromatic or naphthenic hydrocarbons, the PP-derived liquid holds more value as a fuel source compared to the HDPE-derived liquid.

### 3.9. Analysis of char from plastic pyrolysis

#### 3.9.1. X-Ray diffraction analysis.

**Fig 10:**
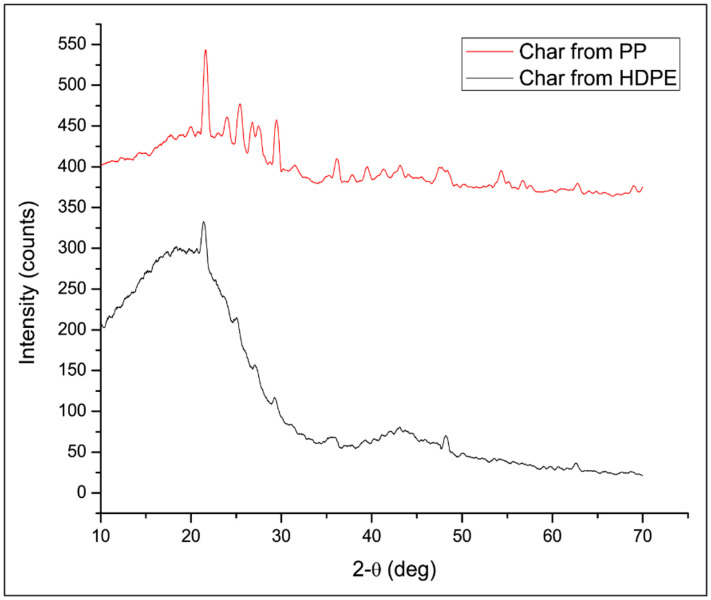
XRD of bio char from PP and HDPE.

X-ray diffraction analysis was conducted to evaluate the crystalline and short-range ordered structures of the char samples obtained from PP and HDPE pyrolysis. As shown in Fig 10, both char samples exhibited a broad diffraction region between approximately 20° and 30° 2θ. This broad band, centered around 21°–24° 2θ, can be assigned to the (002) reflection of disordered carbon, indicating the presence of amorphous or turbostratic carbon structures rather than well-developed graphitic carbon. In addition to this region, a second weak and broad diffraction feature was observed between approximately 40° and 45° 2θ, which may be associated with the (100)/(101) planes of carbonaceous materials. These two broad diffraction regions are commonly associated with poorly ordered carbon structures formed during thermal decomposition and indicate that the produced chars contain partially ordered carbon domains with limited graphitic stacking.

Several minor sharper peaks were also observed in the XRD patterns, particularly in the PP-derived char. These reflections may be related to residual inorganic components, ash-forming species, additives, fillers, pigments, or impurities originally present in the post-consumer plastic waste. Therefore, although the XRD patterns confirm the carbonaceous nature of the produced chars, they also indicate that the chars are not completely pure, fully crystalline, or fully graphitized. The presence of minor crystalline reflections suggests that further purification or activation, such as washing, acid treatment, or controlled thermal activation, may be required if the char is intended for high-purity activated carbon or advanced adsorbent applications.

A comparative assessment of the PP- and HDPE-derived char patterns shows that both chars are mainly carbonaceous and poorly ordered, but they differ in the intensity and sharpness of their diffraction features. The HDPE-derived char is characterized mainly by broad diffraction features in the 20°–30° and 40°–45° 2θ regions, suggesting a predominantly amorphous or turbostratic carbon structure with limited graphitic ordering. In contrast, the PP-derived char shows more pronounced sharper reflections superimposed on the carbon background, particularly in the higher 2θ region. These sharper peaks may indicate the presence of relatively higher crystalline inorganic residues, ash-forming species, fillers, pigments, or additives retained from the original plastic waste. Therefore, the difference between PP- and HDPE-derived chars is not only related to carbon structural ordering but may also be influenced by residual inorganic components associated with the feedstock. Since XRD alone cannot confirm the exact chemical identity of these minor crystalline phases, further analysis such as EDS, XRF, ICP-OES, or ash analysis would be required for precise identification. Overall, HDPE-derived char appears comparatively more amorphous, whereas PP-derived char may contain more detectable crystalline residues and may require further purification for high-value activated carbon applications.

#### 3.9.2. Scanning electron microscopic (SEM) studies.

**Fig 11:**
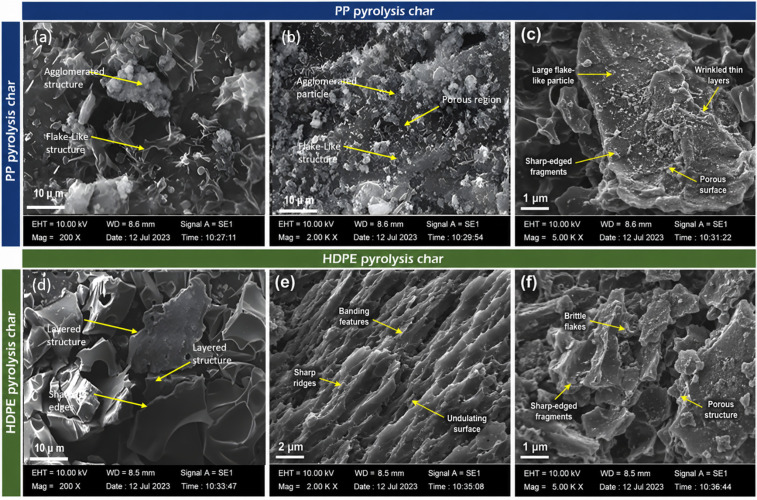
SEM micrographs of char obtained from the pyrolysis of PP and HDPE. (a) PP-derived char showing agglomerated particles (b) PP-derived char exhibiting porous regions, agglomerated particles, and fiber-like structures; (c) PP-derived char displaying large flake-like particles, (d) HDPE-derived char showing layered structures and sharp edges (e) HDPE-derived char illustrating banding features and (f) HDPE-derived char characterized by brittle flakes, porous structures.

The surface morphology of char produced from the pyrolysis of polypropylene (PP) and high-density polyethylene (HDPE) was investigated using scanning electron microscopy (SEM), as shown in Fig 11. The SEM micrographs reveal distinct morphological characteristics for the chars derived from the two polymers, reflecting differences in their thermal degradation behavior and char formation mechanisms.

The PP-derived char (Fig 11a–c) exhibits a heterogeneous carbonaceous structure composed of agglomerated particles, flake-like structures, porous regions, and fiber-like features. At lower magnification (Fig 11a), clusters of agglomerated particles are distributed throughout a matrix of irregular flakes, indicating the formation of a porous carbon framework during pyrolysis. At higher magnification (Fig 11b), interconnected porous regions and fiber-like structures become more evident, suggesting the release of volatile compounds and the development of internal pore networks during thermal decomposition. The micrograph shown in Fig 11c further reveals large flake-like particles, wrinkled thin layers, and sharp-edged fragments. These features indicate localized shrinkage, polymer chain scission, and structural rearrangement during carbonization. The well-developed porous morphology observed in the PP-derived char is advantageous for the impregnation of activating agents and may facilitate its conversion into activated carbon with a high surface area. Such characteristics are desirable for applications in adsorption, water purification, gas treatment, and catalyst support materials.

In contrast, the HDPE-derived char (Fig 11d–f) exhibits a more fragmented and layered morphology. As shown in Fig 11d, the surface is characterized by layered structures with prominent sharp edges, indicating brittle fracture behavior during pyrolysis. At higher magnification (Fig 11e), distinct banding features, sharp ridges, and undulating surface textures are observed, suggesting anisotropic carbon formation and localized thermal stress effects. The micrograph in Fig 11f reveals brittle flakes, porous structures, and sharp-edged fragments, indicating extensive fragmentation and pore development during decomposition. The porous nature of the HDPE-derived char is also favorable for activation processes; however, its morphology appears more irregular and fractured than that of the PP-derived char.

The observed morphological differences between PP- and HDPE-derived chars can be attributed to variations in polymer structure, crystallinity, decomposition kinetics, and volatilization behavior during pyrolysis. While both chars possess porous structures suitable for activation, the PP-derived char exhibits a comparatively more interconnected porous framework, whereas the HDPE-derived char demonstrates greater fragmentation and layered carbon formation. These characteristics suggest that both materials have potential for value-added applications such as activated carbon production, adsorbents, catalyst supports, and carbon-based composite materials. Further optimization of pyrolysis conditions and activation processes may enhance their surface properties and expand their industrial applicability.

### 3.10. Preliminary economic consideration of char production

A preliminary char-yield estimation was carried out to evaluate the possible resource-recovery potential of the solid residue obtained from plastic pyrolysis. Based on the char yields obtained in this study, the amount of char produced from 1 ton of plastic waste would vary depending on the plastic type. For 1 ton of PET, PVC, HDPE, and PP waste, the estimated char production would be approximately 188.5 kg, 343.6 kg, 67.2 kg, and 52.1 kg, respectively. Based on the experimental char yields obtained in this study, a preliminary mass-balance estimation was conducted to calculate the amount of char that could be produced from 1 metric tonne of different plastic wastes. The estimated char production would be approximately 188.5 kg from PET, 343.6 kg from PVC, 67.2 kg from HDPE, and 52.1 kg from PP, while an equal-mass mixed plastic feedstock would produce approximately 162.9 kg of char per tonne (S7 Table in supporting information). Therefore, PVC and PET generated higher char yields, whereas PP and HDPE produced lower char yields but higher liquid-oil fractions.

From an economic perspective, the char can contribute to process feasibility in two ways. First, it can be used as a supplementary solid fuel or heat source, thereby reducing external energy demand during pyrolysis. Second, after appropriate purification and activation, the carbon-rich char may be upgraded into activated carbon or adsorbent material for environmental applications such as wastewater treatment, gas adsorption, or pollutant removal. However, the XRD results indicate that the char may contain residual inorganic matter, ash-forming components, additives, or impurities, especially in PP-derived char. Therefore, additional post-treatment steps such as washing, acid treatment, or thermal/chemical activation may be required before high-value applications.

The preliminary economic feasibility of char utilization depends on the plastic feedstock, char yield, purity, activation cost, and local market demand. For example, if 1 ton of mixed plastic waste containing equal proportions of PET, PVC, HDPE, and PP is considered, the average char yield would be approximately 16.29%, equivalent to about 162.9 kg of char per ton of mixed plastic waste. This indicates that char recovery can provide an additional value stream along with pyrolysis oil and gas, although the main economic product in the present study remains the liquid fuel fraction, particularly from PP and HDPE. Further techno-economic analysis, including energy balance, activation cost, purification cost, and market evaluation, is recommended for large-scale feasibility assessment.

## 4. Conclusion

This study has successfully characterized the physical and chemical properties of pyrolysis oil derived from waste plastics, comparing them with those of conventional fuels. The pyrolytic liquids obtained from the process, which consist of a mixture of paraffins, olefins, and aromatic compounds, exhibit a dark brown color and an acrid odor. With a moderate gross calorific value (GCV) of approximately 25–27 MJ/kg, these liquids demonstrate significant potential as replacements for traditional liquid fuels. The pyrolysis oils, with their low ash content, show promise for use in industrial furnaces, power plants, and boilers. The similarities between the distillation curves of the pyrolysis oil and conventional diesel or crude oil further validate their potential as alternative fuels. Fractional distillation yielded a substantial quantity of pyro-oil within the 81–140 °C temperature range, a fraction that could be optimized for fuel use. The study also highlighted that the density, viscosity, GCV, carbon, and hydrogen contents of the pyrolysis liquids are comparable to those of commercial diesel fuels, making them suitable for blending with petroleum refinery streams after processes such as desulfurization and dehydrogenation. Additionally, the liquid fractions within the 150–300 °C range show potential for use as diesel fuel or heating oil. Notably, the pyrolytic liquids are enriched with olefins, including limonene and mild aromatics, which possess greater market value as chemical feedstocks than as fuel. Moreover, the naphtha fraction derived from the oil demonstrated a higher octane number than petroleum naphtha, indicating its suitability as an environmentally friendly fuel source. The XRD and SEM analyses of the resulting char revealed a porous structure with uniformly distributed carbon, suggesting its potential for various industrial applications, including the production of activated carbon.

## Supporting information

S1 Fig Graphical abstract. (TIF)

S1 Table FT-IR spectra of raw PP. (DOCX)

S2 Table FT-IR spectra of PP pyrolysis oil. (DOCX)

S3 Table FT-IR spectra of Raw HDPE. (DOCX)

S4 Table FT-IR spectra of HDPE pyrolysis oil. (DOCX)

S5 Table The probable compound present in the crude oil by GC-MS data. (DOCX)

S6 Table The probable compound present in the distillate product by GC-MS data. (DOCX)

S7 Table Estimated char production from 1 metric tonne of plastic waste based on experimental char yields. (DOCX)

## References

[1] Mukherjee A, Ruj B, Sadhukhan AK, Gupta P, Chakrabortty S, Chatterjee R, et al. Eco-synthesis, characterization and application of waste plastics pyrolysis char in arsenic removal from contaminated water: An integrated circular framework with parametric response surface methodology optimization-cum-artificial neural network model. Journal of Environmental Chemical Engineering. 2024;12(1):111824. doi: 10.1016/j.jece.2023.111824

[2] Islam MK, Khatun MS, Arefin MA, Islam MR, Hassan M. Waste to energy: An experimental study of utilizing the agricultural residue, MSW, and e-waste available in Bangladesh for pyrolysis conversion. Heliyon. 2021;7(12):e08530. doi: 10.1016/j.heliyon.2021.e08530 34917811 PMC8665337

[3] Muntasir Shovon S, Ahamed Akash F, Abdur Rahman Md, Rahman W, Chakraborty P, Uddin Monir M, et al. The potential for sustainable waste management and energy recovery in Bangladesh: A review. Sustainable Energy Technologies and Assessments. 2024;64:103705. doi: 10.1016/j.seta.2024.103705

[4] Kibria MdG, Paul UK, Hasan A, Mohtasim MdS, Das BK, Mourshed M. Current prospects and challenges for biomass energy conversion in Bangladesh: Attaining sustainable development goals. Biomass and Bioenergy. 2024;183:107139. doi: 10.1016/j.biombioe.2024.107139

[5] Mohan I, Yao Z, Das AK, Prakash R, Kumar S. Performance, emission and combustion characteristics of liquid fuel produced through catalytic co-pyrolysis of waste LDPE and Pongamia pinnata seeds: An experimental investigation in CI engine. Results in Engineering. 2023;20:101499. doi: 10.1016/j.rineng.2023.101499

[6] Hussain I, Ganiyu SA, Alasiri H, Alhooshani K. A state-of-the-art review on waste plastics-derived aviation fuel: Unveiling the heterogeneous catalytic systems and techno-economy feasibility of catalytic pyrolysis. Energy Conversion and Management. 2022;274:116433. doi: 10.1016/j.enconman.2022.116433

[7] Mohanty A, Ajmera S, Chinnam S, Kumar V, Mishra RK, Acharya B. Pyrolysis of waste oils for biofuel production: An economic and life cycle assessment. Fuel Communications. 2024;18:100108. doi: 10.1016/j.jfueco.2024.100108

[8] Marchetti L, Guastaferro M, Annunzi F, Tognotti L, Nicolella C, Vaccari M. Two-stage thermal pyrolysis of plastic solid waste: Set-up and operative conditions investigation for gaseous fuel production. Waste Manag. 2024;179:77–86. doi: 10.1016/j.wasman.2024.03.011 38461626

[9] Patil PD, Gude VG, Deng S. Biodiesel Production from Jatropha Curcas, Waste Cooking, and Camelina Sativa Oils. Ind Eng Chem Res. 2009;48(24):10850–6. doi: 10.1021/ie901146c

[10] Abdelfatah M, Kiros Y, Abu Elala R. Biodiesel production from waste cooking oil using different heterogeneous catalysts support on alumina. Petrol Petrochem Eng J. 2017;1(6):1–7. doi: 10.23880/ppej-16000134

[11] Chowdhury GW, Koldewey HJ, Duncan E, Napper IE, Niloy MNH, Nelms SE, et al. Plastic pollution in aquatic systems in Bangladesh: A review of current knowledge. Sci Total Environ. 2021;761:143285. doi: 10.1016/j.scitotenv.2020.143285 33172641

[12] Al-Fatesh AS, AL-Garadi NYA, Osman AI, Al-Mubaddel FS, Ibrahim AA, Khan WU, et al. From plastic waste pyrolysis to Fuel: Impact of process parameters and material selection on hydrogen production. Fuel. 2023;344:128107. doi: 10.1016/j.fuel.2023.128107

[13] Dizge N, Aydiner C, Imer DY, Bayramoglu M, Tanriseven A, Keskinler B. Biodiesel production from sunflower, soybean, and waste cooking oils by transesterification using lipase immobilized onto a novel microporous polymer. Bioresour Technol. 2009;100(6):1983–91. doi: 10.1016/j.biortech.2008.10.008 19028094

[14] Akgül A, Palmeiro-Sanchez T, Lange H, Magalhaes D, Moore S, Paiva A, et al. Characterization of tars from recycling of PHA bioplastic and synthetic plastics using fast pyrolysis. J Hazard Mater. 2022;439:129696. doi: 10.1016/j.jhazmat.2022.129696 36104917

[15] Kumar Mishra R, Mohanty K. Co-pyrolysis of waste biomass and waste plastics (polystyrene and waste nitrile gloves) into renewable fuel and value-added chemicals. Carbon Resources Conversion. 2020;3:145–55. doi: 10.1016/j.crcon.2020.11.001

[16] Luyben WL. Distillation column pressure selection. Separation and Purification Technology. 2016;168:62–7. doi: 10.1016/j.seppur.2016.05.015

[17] Tengku Hassan TNA, Shariff AM, Mohd Pauzi MM, Khidzir MS, Surmi A. Insights on Cryogenic Distillation Technology for Simultaneous CO2 and H2S Removal for Sour Gas Fields. Molecules. 2022;27(4):1424. doi: 10.3390/molecules27041424 35209212 PMC8879961

[18] Li Y, Nahil MA, Williams PT. Pyrolysis-catalytic steam reforming of waste plastics for enhanced hydrogen/syngas yield using sacrificial tire pyrolysis char catalyst. Chemical Engineering Journal. 2023;467:143427. doi: 10.1016/j.cej.2023.143427

[19] Sharypov VI, Beregovtsova NG, Kuznetsov BN, Membrado L, Cebolla VL, Marin N, et al. Co-pyrolysis of wood biomass and synthetic polymers mixtures. Part III: Characterisation of heavy products. Journal of Analytical and Applied Pyrolysis. 2003;67(2):325–40. doi: 10.1016/s0165-2370(02)00071-2

[20] Sørum L, Grønli MG, Hustad JE. Pyrolysis characteristics and kinetics of municipal solid wastes. Fuel. 2001;80(9):1217–27. doi: 10.1016/s0016-2361(00)00218-0

[21] DEMIRBAS A. Pyrolysis of municipal plastic wastes for recovery of gasoline-range hydrocarbons. Journal of Analytical and Applied Pyrolysis. 2004. doi: 10.1016/s0165-2370(04)00029-4

[22] Achilias DS, Roupakias C, Megalokonomos P, Lappas AA, Antonakou EV. Chemical recycling of plastic wastes made from polyethylene (LDPE and HDPE) and polypropylene (PP). J Hazard Mater. 2007;149(3):536–42. doi: 10.1016/j.jhazmat.2007.06.076 17681427

[23] Saad JM, Williams PT. Pyrolysis-Catalytic-Dry Reforming of Waste Plastics and Mixed Waste Plastics for Syngas Production. Energy Fuels. 2016;30(4):3198–204. doi: 10.1021/acs.energyfuels.5b02508

[24] Adrados A, de Marco I, Caballero BM, López A, Laresgoiti MF, Torres A. Pyrolysis of plastic packaging waste: A comparison of plastic residuals from material recovery facilities with simulated plastic waste. Waste Manag. 2012;32(5):826–32. doi: 10.1016/j.wasman.2011.06.016 21795037

[25] López A, de Marco I, Caballero BM, Laresgoiti MF, Adrados A. Influence of time and temperature on pyrolysis of plastic wastes in a semi-batch reactor. Chemical Engineering Journal. 2011;173(1):62–71. doi: 10.1016/j.cej.2011.07.037

[26] Williams PT, Slaney E. Analysis of products from the pyrolysis and liquefaction of single plastics and waste plastic mixtures. Resources, Conservation and Recycling. 2007;51(4):754–69. doi: 10.1016/j.resconrec.2006.12.002

[27] Yasin G, Bhanger MI, Ansari TM, Naqvi S, Talpur FN. Quality of commercial high speed diesel and its environmental impact. Journal of Petroleum Technology and Alternative Fuels. 2012;3(3):29–35. doi: 10.5897/JPTAF11.031

