# Supplementary figures and images for: Sustainable conversion of waste plastics to biofuel: Process insights and fuel characteristics
Source: PLoS One. 2026 Jul 31;21(7):e0354825. doi: 10.1371/journal.pone.0354825 (PMC13426997; doi:10.1371/journal.pone.0354825)

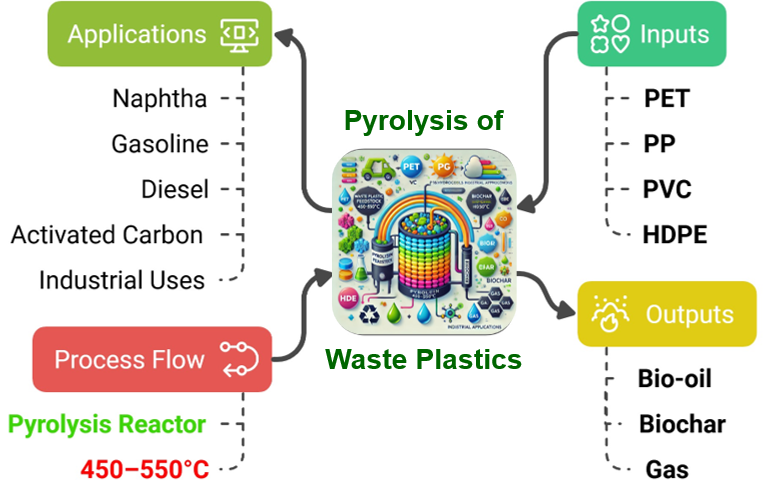

Supplement: S1 Fig — (TIF) [file pone.0354825.s001.tif]
