# Supplementary material for: Sustainable conversion of waste plastics to biofuel: Process insights and fuel characteristics
Source: PLoS One. 2026 Jul 31;21(7):e0354825. doi: 10.1371/journal.pone.0354825 (PMC13426997; doi:10.1371/journal.pone.0354825)
Supplement: S1 Table — (DOCX) [file pone.0354825.s002.docx]

**Supporting Information**

**Sustainable conversion of waste plastics to biofuel: process insights and fuel characteristics**

| **Table S1. FT-IR spectra of raw PP.** |
| --- |
| \| **Functional group** \| **Theoretical frequency range (cm-1)** \| **Actual peak** \| **Class of compounds** \| \| --- \| --- \| --- \| --- \| \| C-H stretching \| near 3000 \| 2904.8 \| Alkanes \| \| CH2 and CH3 bending \| 1475-1365 \| 1419.61 \| \| C=C stretching \| 1600-1450 \| 1585.49 \| Aromatic ring \| \| C=C (oop) \| 900-690 \| 709.8 \| \| C-O \| 1250–1300 \| 1280.73 \| Carboxylic acids \| \| C-H bending (oop) \| 1000-650 \| 941.26 \| Alkenes \| \| O=C=O Stretching \| 2400-2000 \| 2345.44 \| Carbon dioxide \| |
