# Supplementary material for: Sustainable conversion of waste plastics to biofuel: Process insights and fuel characteristics
Source: PLoS One. 2026 Jul 31;21(7):e0354825. doi: 10.1371/journal.pone.0354825 (PMC13426997; doi:10.1371/journal.pone.0354825)
Supplement: S2 Table — (DOCX) [file pone.0354825.s003.docx]

**Supporting Information**

**Sustainable conversion of waste plastics to biofuel: process insights and fuel characteristics**

| **Table S3. FT-IR spectra of Raw HDPE.** |
| --- |
| \| **Functional group** \| **Theoretical frequency range (cm-1)** \| **Actual peak** \| **Class of compounds** \| \| --- \| --- \| --- \| --- \| \| CH2 and CH3 bending \| 1475-1365 \| 1342.46 \|  \| \| C-H stretching \| greater 3000 \| 3059.10 \| Alkenes \| \| C-H bending (oop) \| 1000-650 \| 725.23 \| \| N-H bend \| 1640-1560 \| 1620.21 \| Secondary amines \| \| O=C=O Stretching \| 2400-2000 \| 2341.58 \| Carbon dioxide \| \| O-H \| 3200-3400 \| 3336.85 \| Alcohols, Phenols \| |
