# Supplementary material for: Sustainable conversion of waste plastics to biofuel: Process insights and fuel characteristics
Source: PLoS One. 2026 Jul 31;21(7):e0354825. doi: 10.1371/journal.pone.0354825 (PMC13426997; doi:10.1371/journal.pone.0354825)
Supplement: S4 Table — (DOCX) [file pone.0354825.s005.docx]

**Supporting Information**

**Sustainable conversion of waste plastics to biofuel: process insights and fuel characteristics**

| **Table S4. FT-IR spectra of HDPE pyrolysis oil.** |
| --- |
| \| **Functional group** \| **Theoretical frequency range (cm-1)** \| **Actual peak** \| **Class of compounds** \| \| --- \| --- \| --- \| --- \| \| C-H stretching \| near 3000 \| 2935.66 \| Alkanes \| \| C=C stretching \| 1600-1450 \| 1411.89 \| Aromatic rings \| \| C=C (oop) \| 900-690 \| 709.8 \| \| C=O stretch \| 1850-1650 \| 1693.5 \| Carbonyl group \| \| O-H \| 3200-3400 \| 3340.71 \| Alcohols, Phenols \| \| CH2 and CH3 bending \| 1475-1365 \| 1411.89 \| Alkanes \| \| O=C=O Stretching \| 2400-2000 \| 2337.72 \| Carbon dioxide \| |
