# Supplementary material for: Sustainable conversion of waste plastics to biofuel: Process insights and fuel characteristics
Source: PLoS One. 2026 Jul 31;21(7):e0354825. doi: 10.1371/journal.pone.0354825 (PMC13426997; doi:10.1371/journal.pone.0354825)
Supplement: S6 Table — (DOCX) [file pone.0354825.s007.docx]

**Supporting Information**

**Sustainable conversion of waste plastics to biofuel: process insights and fuel characteristics**

| **Table S6. The probable compound present in the distillate product by GC-MS data.** |
| --- |
| 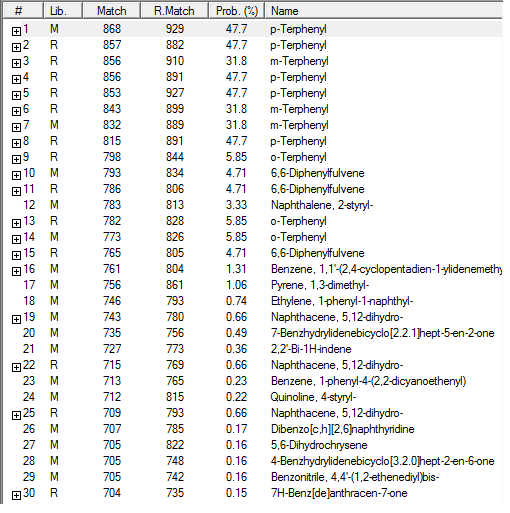 |
