# Supplementary material for: Sustainable conversion of waste plastics to biofuel: Process insights and fuel characteristics
Source: PLoS One. 2026 Jul 31;21(7):e0354825. doi: 10.1371/journal.pone.0354825 (PMC13426997; doi:10.1371/journal.pone.0354825)
Supplement: S7 Table — (DOCX) [file pone.0354825.s008.docx]

**Supporting Information**

**Sustainable conversion of waste plastics to biofuel: process insights and fuel characteristics**

**Table S7. Estimated char production from 1 metric tonne of plastic waste based on experimental char yields.**

| **Plastic type** | **Char yield (%)** | **Estimated char from 1 ton plastic waste** |
| --- | --- | --- |
| PET | 18.85 | 188.5 kg |
| PVC | 34.36 | 343.6 kg |
| HDPE | 6.72 | 67.2 kg |
| PP | 5.21 | 52.1 kg |
| Equal-mass mixed plastic | 16.29 | 162.9 kg |
